# Ethyl Acetate Fraction from* Hedyotis diffusa* plus* Scutellaria barbata* Exerts Anti-Inflammatory Effects by Regulating miR-155 Expression and JNK Signaling Pathway

**DOI:** 10.1155/2018/3593408

**Published:** 2018-03-14

**Authors:** Yuan Xu, Xiao-Xia Chen, Yi-Xin Jiang, Dan-Dan Zhang

**Affiliations:** ^1^Institute of Interdisciplinary Integrative Medicine Research, Shanghai University of Traditional Chinese Medicine, Shanghai 201203, China; ^2^School of Pharmacy, Shanghai University of Traditional Chinese Medicine, Shanghai 201203, China

## Abstract

*Hedyotis diffusa *Willd and* Scutellaria barbata *D. Don (HDSB) were the core couplet in medicines that were commonly used for the purpose of anti-inflammation and anticancer treatments in China. However, biological properties of this couplet have not been fully elucidated. In this study, we screened fractions of HDSB for their anti-inflammatory activities and explored pertinent molecular mechanisms on murine macrophage RAW264.7 cell model. Ethyl acetate fraction from the aqueous extract of the couplet at equal weight ratio (EA11) showed the strongest inhibition of the nitrite accumulation in supernatant of RAW264.7 cells stimulated with lipopolysaccharide (LPS)/interferon-*γ* (IFN-*γ*). In addition, EA11 inhibited iNOS and IL-1*β* expression in a concentration-dependent manner while promoting the expression of HO-1 and PPAR-*γ*. Anti-inflammatory capability is most likely facilitated by its inhibitory effect on JNK signaling pathway and miR-155 expression. This study suggests that EA11 may be represented as a potential anti-inflammatory therapeutic candidate.

## 1. Introduction

Chronic and uncontrolled inflammation plays a critical role in various pathological conditions, including autoimmune disorders, cardiovascular diseases, and cancers [1]. LPS, a component from the outer membranes in Gram-negative bacteria, can induce macrophages with a serial of inflammatory cascades characterized by the nitric oxide (NO) accumulation and release of inflammatory mediators through the activation of the MAPK signaling pathway. NO, a gaseous free radical, is produced by nitric oxide synthase (NOS) which has three subfamilies including eNOS, nNOS, and inducible nitric oxide synthase (iNOS). iNOS responses are responsible for high level production of NO during pathological inflammation [2].

Chronic inflammation may be regulated by diverse mechanisms and molecules. For example, heme oxygenase- (HO-1), an inducible enzyme for heme degradation, can decrease the expressions of proinflammatory mediators in stimulated macrophages and overexpression of HO-1 is often associated with iNOS suppression [3]. Under the condition of inflammation, MAPKs including JNK, p38, and ERK have also been found to regulate the production of proinflammatory cytokines such as iNOS and IL-1*β* [4]. Moreover, recent study showed that level of miRNA-155 increases upon LPS stimulation in macrophages and was considered as a potential regulator of inflammation [5].

*Hedyotis diffusa *Willd (HD) belongs to the Rubiaceae family and is widely distributed in southern China and other Asian countries. Accumulating pieces of evidence suggest that HD possesses potent anti-inflammation and cancer activities [6–9].* Scutellaria barbata* D. Don (SB) contains flavonoids and scutebarbatines as its mainly active components and has been evaluated for its bioactivity in immune diseases and cancer [10–12]. In fact,* Hedyotis diffusa* and* Scutellaria barbata *(HDSB) were often used as common herb pairs (couplet medicines) for the treatment of inflammation and kinds of cancer in China [13, 14]. However, the effect of HDSB on inflammation is unclear.

The objective of this study is to clarify anti-inflammatory mechanisms of active fraction from HDSB. Our results indicate that EA11, the ethyl acetate fraction extracted at equal ratio of HD and SB, showed the strongest inhibition of NO production among all fractions that were tested. With the aid of ultra performance liquid chromatography (UPLC), we characterized the chemical constituents in EA11. EA11 suppressed the production of proinflammatory mediators (iNOS and IL-1*β*) while upregulating anti-inflammatory mediators (HO-1 and PPAR-*γ*). We present pieces of evidence that EA11 exerts its anti-inflammatory activity through the regulation of miR-155 expression and JNK signaling pathway.

## 2. Materials and Methods

### 2.1. Reagents

LPS (Escherichia coli, serotype 055:B5), L-N^6^-(1-Iminoethyl)lysine hydrochloride (L-NIL), N-(1-naphthyl) ethylenediamine dihydrochloride, and 3-[4,5-dimethylthiazol-2-yl]-2,5-diphenyltetrazolium bromide (MTT) were obtained from Sigma-Aldrich (St. Louis, MO, USA); murine recombinant IFN*γ* was purchased from Millipore (MA, USA). Trizol Reagent was obtained from Invitrogen (Carlsbad, CA). Takara SYBR kit and Oligo(dT) were obtained from Shanghai Invitrogen (Shanghai, China). Chemical standard compounds including 4′-hydroxyacetophenone, scutellarin, luteolin, and apigenin were kindly provided by Professor Wei-Dong Zhang (Shanghai University of Traditional Chinese Medicine); anti-iNOS was obtained from Cayman Chemical (Ann Arbor, MI, USA); anti-HO-1 and anti-PPAR-*γ* were obtained from Abcam (Cambridge, UK); anti-SAPK/JNK, anti-p-SAPK/JNK, anti-ERK1/2, anti-p-ERK1/2, anti-p38/MAPK, anti-p-p38/MAPK, and anti-*β*-actin were obtained from Cell Signaling Technology (Boston, MA, USA); nitrocellulose (NC) membranes were acquired from Millipore (Bedford, MA, USA); TaqMan MicroRNA primers, transcription kit, and universal PCR master mix were obtained from Applied Biosystems (ABI, Foster City, CA, USA). All other chemicals were of analytical grade.

### 2.2. Preparation of Extract and Isolation of Fractions

*Hedyotis diffusa Willd *and* Scutellaria barbata D. Don *were purchased from Yang He Tang Co. (Zhangjiang High-Tech Park, Shanghai, China) and identified by Shanghai Institute for Food and Drug Control (SIFDC). The dried herbs were extracted in 1 : 2, 2 : 1, 1 : 1 weight ratio with filtered water to gain three aqueous extracts (1 : 2 W; 2 : 1 W; 1 : 1 W) using a rotary vacuum evaporator (Buchi, Switzerland). Then, these extracts were suspended in distilled water and successively partitioned with petroleum ether, ethyl acetate, *n*-butanol, and water separately to gain twelve totally different fractions. These extracts and fractions were dissolved in DMSO to 20 mg/ml and stored at −20°C for required further concentrations. The final concentration of DMSO in the cell culture studies was controlled at ≤0.5%.

### 2.3. Cell Culture

The murine macrophage RAW264.7 cell line was obtained from American Type Culture Collection (ATCC, Rockville, MD, USA). Macrophages were cultured in RPMI 1640 medium supplemented with 10% fetal bovine serum (FBS) in a humidified incubator at 37°C with 5% CO_2_.

### 2.4. Ultra Performance Liquid Chromatography Assay

EA11 standardization was performed using UPLC fingerprinting with chemical standard compounds. Standard solutions were prepared by dissolving 1 mg/mL of each marker component in 100% methanol. EA11 powder was weighed accurately and dissolved in methanol at a concentration of 5 mg/mL for analysis. UPLC analysis was performed on a Waters ACQUITY UPLC system (Waters corporation, Milford, MA, USA) using Column of Waters ACQUITY UPLC® HSS T3 (1.8 *μ*m, 2.1*∗*100 mm), equipped with a binary solvent delivery system and an autosampler. And the detector scanned from 200 to 400 nm. The mobile phase consisted of gradient mixture of acetonitrile (solvent A) and 0.1% formic acid in water (solvent B) at a flow rate of 0.4 ml/min. A gradient elution was as follows: 0–9 min, 5%–53% A (v/v) for 0–9 min; 9-10 min, 53%–100% A (v/v); 10–10.1 min, 100%–5% A (v/v); 10.1–13 min, 5% A (v/v) in order to reequilibrate the column at initial conditions.

### 2.5. Measurement of NO Accumulation

NO accumulation was measured in the cell culture medium using Griess reagent. Briefly, RAW264.7 cells were treated with different extracts and fractions at 10, 50, 100, and 200 *μ*g/ml followed by LPS (100 ng/ml) plus IFN-*γ* (10 U/ml) for 24 h. L-NIL, which is the inhibitor of iNOS, was used as a positive control in this study (50 *μ*M). Following the collection of the supernatant, each sample (100 *μ*l) was mixed with the same volume of Griess reagent and incubated at room temperature for 10 min. The absorbance was read at 540 nm using a microplate reader. Nitrate standard curve was used to calculate the nitrite production in the samples. The percentage inhibition is evaluated using the formula: {1 − [nitrite amount of fraction-treated/nitrite amount of vehicle]}  × 100.

### 2.6. Cell Viability Assay

Cell viability was determined by MTT assay. RAW264.7 cells were seeded at a density of 1 × 10^4^ cells/well and cultured overnight in a 37°C incubator. Cells were treated with vehicle (DMSO) or 25–50 *μ*g/ml EA11 for 24 h. 10 *μ*l MTT solution [5 mg/ml in phosphate-buffered saline (PBS), pH = 7.4] was added to each well. Cells were incubated at 37°C for a further 4 h. Formazan was dissolved and the absorbance at 490 nm was read using a plate reader (Molecular Devices, CA, USA). The morphological features of RAW264.7 cells in each treated group were also observed using microscope. The relative viability was calculated when the absorbance of control group was considered 100% of viability.

### 2.7. Total RNA Isolation and qPCR

Total cellular RNA was extracted by Trizol Reagent according to the manufacturer's procedure. The concentration of RNA was measured and adjusted to 500 ng/*μ*l. Total RNA was converted to cDNA using RT reagent. The oligonucleotide primers for qRT-PCR used with cDNA are listed in Table 2 as previously described [15]. The reactions were conducted in triplicate with a total volume of 20 *μ*L, comprised of 0.3 *μ*M of each primer, 10 *μ*L of SYBR Green Master, and 2 *μ*L of template DNA. PCR was used to detect IL-1*β*, iNOS, HO-1, and GAPDH following 40 cycles of 95°C for 15 s and 60°C for 1 min. Amplification and analyses were performed using ABI 7500 Real-Time PCR System. Samples were compared using the relative CT method. Individual transcripts in each sample were normalized to GAPDH mRNA.

### 2.8. miRNA Determination

Total RNA of each group was extracted by Trizol Reagent according to manufacturer's instructions. Reverse transcription reaction was performed with specific miRNA primers by miScript II RT kit (ABI, Foster City, CA, USA). The expression of miR-155 was determined by miScript SYBR Green PCR Kit (ABI, Foster City, CA, USA). Real-time PCR amplification was done with a ABI 7500 machine using standard conditions. Relative miRNA expressions are normalized to the endogenous control U6.

### 2.9. Western Blotting

RAW264.7 cells were treated with vehicle or pretreated with 25 or 50 *μ*g/ml EA11 in FBS free DMEM medium for 24 h. Cells were subsequently incubated for 30 min, 6 h, and 24 h with LPS (100 ng/ml) plus IFN-*γ* (10 U/ml). Total protein for western blotting was extracted from RAW264.7 cells using radioimmunoprecipitation assay (RIPA) buffer (Beyotime Technology, Jiangsu, China) with phosphatase inhibitor cocktails (Roche, Basel, Switzerland), by incubation on ice for 30 min and subsequent centrifugation at 12,000 ×g (4°C, 15 min). Protein concentration of samples was determined in the supernatants by the BCA protein assay kit (Beyotime Technology, Jiangsu, China). Proteins (30 *μ*g) were separated by 4–12% SDS gel by SDS-polyacrylamide gel electrophoresis (PAGE) and transferred to nitrocellulose membranes for 2 h. After the blocking with 5% skim milk and incubating at room temperature for 1 h, the membranes were incubated overnight at 4°C with the following primary antibodies: anti-iNOS, anti-HO-1, anti-PPAR-*γ*, anti-SAPK/JNK, anti-p-SAPK/JNK, anti-ERK1/2, anti-p-ERK1/2, anti-p38/MAPK, anti-p-p38/MAPK, and anti-*β*-actin. Membranes were then washed with PBS-Tween-20 solution (PBS-T) and incubated with 1 : 1,000–2000 diluted horseradish peroxidase- (HRP-) conjugated goat anti-rabbit IgG at room temperature for 1 h. Blots were developed using ECL Western Blotting Detection Reagent (Millipore, Bedford, MA, USA). Protein bands were visualized using the Tanon imaging system (Tanon, shanghai, China) and the band density was semiquantified using the Tanon Program.

### 2.10. Statistical Analysis

Statistical tests were performed using SPSS version 12.1 (SPSS, Inc., Chicago, IL, USA). All result values are presented as the mean ± standard deviation (SD) from at least three independent experiments. Statistical analysis was performed by Student's* t*-test or one way ANOVA to determine the statistical significance for these experiments.

## 3. Results

### 3.1. EA11 Exhibits Potent Anti-Inflammatory Effect in Screening on Cell Inflammatory Model

To identify ingredients in HDSB that exhibited anti-inflammatory properties, we analyzed effect of various extracts from HDSB on the production of NO by RAW264.7 cells under the stimulation of LPS/IFN*γ*. Griess reaction assay showed that EA11 displayed the lowest IC_50_ (27.74 *μ*g/ml) among all the extracts and fractions (Table 1), which was even more potent than L-NIL, a commonly used iNOS inhibitor that reduced 35.2% of NO production at 50 *μ*M.

### 3.2. EA11 Inhibit LPS/IFN*γ*-Induced NO Production without Significant Cytotoxicity

**Figure 1:**
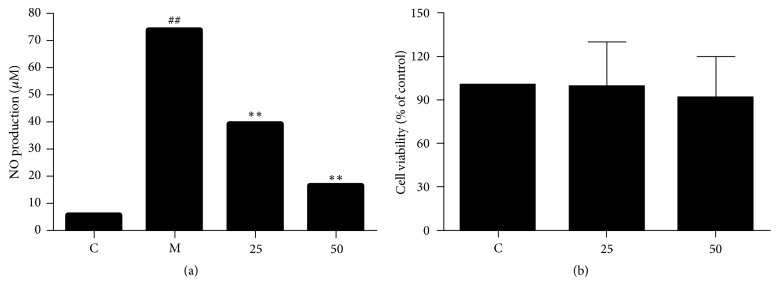
*Effect on NO production and toxicity of EA11.* (a)* NO production* was measured by Griess reaction following incubation with model (LPS/IFN-*γ*) and control (vehicle control) group, 25 or 50 *μ*g/ml EA11 for 24 h. (a) Cell viability of RAW264.7 cells treated with EA11 was determined by MTT assay. Values are presented as the mean ± standard deviation from three replicates. C: vehicle control; M: model group (LPS/IFN*γ* stimulation); EA11: ethyl acetate fraction from 1 : 1 W; ^##^*P* < 0.01 versus C group; ^*∗∗*^*P* < 0.01 versus M group.

We repeated the Griess reaction assay of EA11 at the dosage of 25 and 50 *μ*g/ml, and EA11 displayed nitrite (the steady production of NO) inhibition in a dose-dependent manner (Figure 1(a)). We next examined the effect of EA11 on cell viability by treating RAW264.7 cells with 25 and 50 *μ*g/ml EA11 for 24 h. MTT assay showed that EA11 even at the concentration of 50 *μ*g/ml, which is twice as much as IC_50_, did not elicit significant cytotoxicity to cells (Figure 1(b)). Moreover, observation under microscope also did not find differences in morphological features between EA11-treated and untreated cells. These results indicate that EA11 at dose capable of suppressing nitrite production does not affect cell viability.

### 3.3. EA11 Suppresses the Expression of iNOS in Activated Macrophages

**Figure 2:**
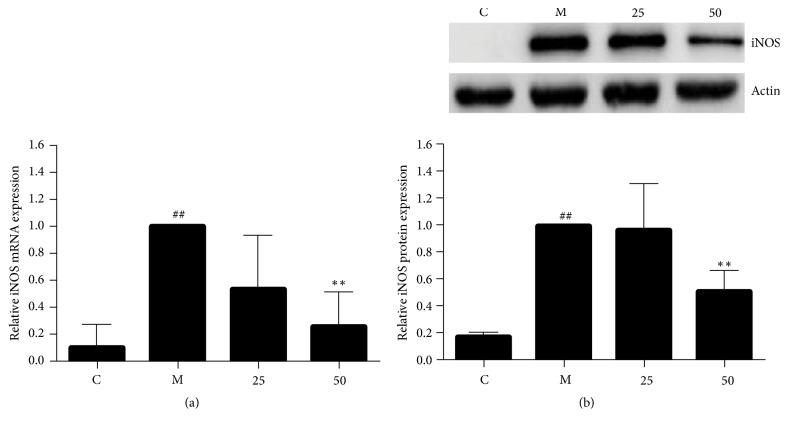
*Analysis of the gene and protein expression of iNOS.* Following treatment of RAW264.7 cells with M (LPS/IFN*γ* stimulation) and C (vehicle control), 25 or 50 *μ*g/ml of EA11, (a) iNOS mRNA levels were assessed by qRT-PCR and (b) iNOS protein levels were assessed by western blot. Western blot data show a representative blot out of three. Values are presented as the mean ± standard deviation of three replicates. Asterisks indicate significant differences compared to model cells. ^##^*P* < 0.01 versus C group; ^*∗∗*^*P* < 0.01 versus M group.

The increase of NO production during inflammation is largely due to the induction of iNOS. We thus investigated the possibility that EA11 blocked NO production by inhibiting iNOS. To test this possibility, we determined the effect of EA11 on iNOS expression in stimulated RAW264.7 cells. qRT-PCR revealed a significant decrease in the level of iNOS mRNA (Figure 2(a)) while western blotting showed that EA11 was able to reduce the abundance of iNOS protein (Figure 2(b)). These results support the notion that EA11 exerts its anti-inflammatory activity by inhibiting iNOS expression.

### 3.4. EA11 Regulated the Expression of Inflammatory Mediators

**Figure 3:**
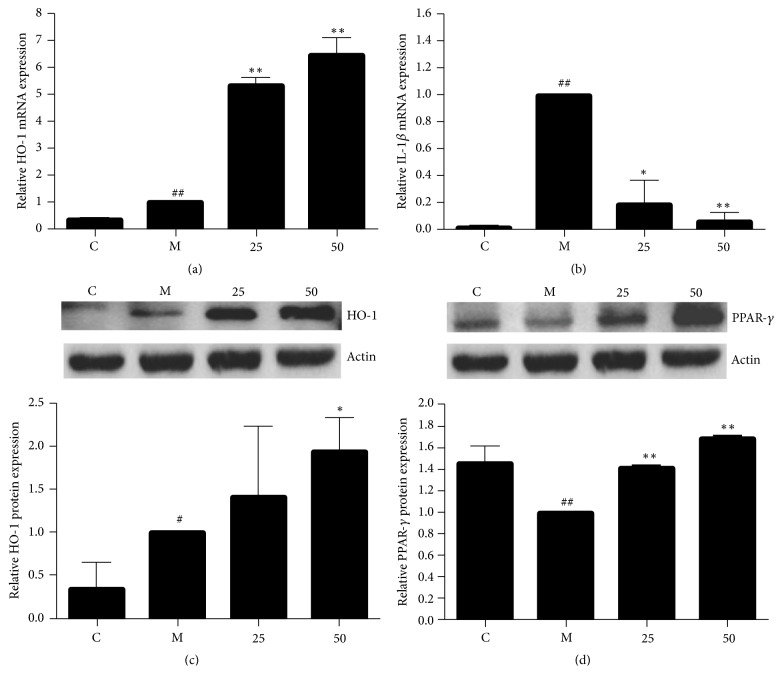
*Analysis of pro- and anti-inflammatory mediators expression.* Following treatment of RAW264.7 cells with M (LPS/IFN*γ* stimulation) and C (vehicle control), 25 or 50 *μ*g/ml of EA11, IL-1*β* (a) and HO-1 (b) mRNA levels were assessed by qRT-PCR and HO-1 (c) and PPAR-*γ* (d) protein levels were assessed by western blot. Western blot data show a representative blot out of three. Values are presented as the mean ± standard deviation from three replicates. Asterisks indicate significant differences compared to model cells. ^##^*P* < 0.01 versus C group; ^#^*P* < 0.05 versus C group; ^*∗*^*P* < 0.05 versus M group; ^*∗∗*^*P* < 0.01 versus M group.

HO-1, the rate-limiting enzyme in the process of free heme degradation, has been shown as a negative regulator of inflammatory responses because the products of free heme degradation inhibit the expression of various proinflammatory mediators. We thus investigated the effect of EA11 on expression of HO-1 in LPS/IFN*γ*-activated RAW264.7 cells by qRT-PCR. Although level of HO-1 mRNA significantly increased upon the stimulation of LPS/IFN*γ*, EA11 further augmented the expression of HO-1 (Figure 3(a)). The increase of HO-1 abundance was also confirmed by western blotting using specific antibody (Figure 3(c)). In addition, we also observed dose-dependent increase in the level of PPAR-*γ*, an anti-inflammatory mediator, by EA11 (Figure 3(d)). In contrast, EA11 prevented the upregulation of IL-1*β*, a proinflammatory factor, by LPS/IFN*γ* stimulation in RAW264.7 cells (Figure 3(b)). These results suggest that EA11 can also suppress inflammatory response by simultaneously upregulating anti-inflammatory mediators and downregulating inflammatory promotor expression.

### 3.5. Effects of EA11 on MAPK Signaling Pathway and miR-155 Expression

**Figure 4:**
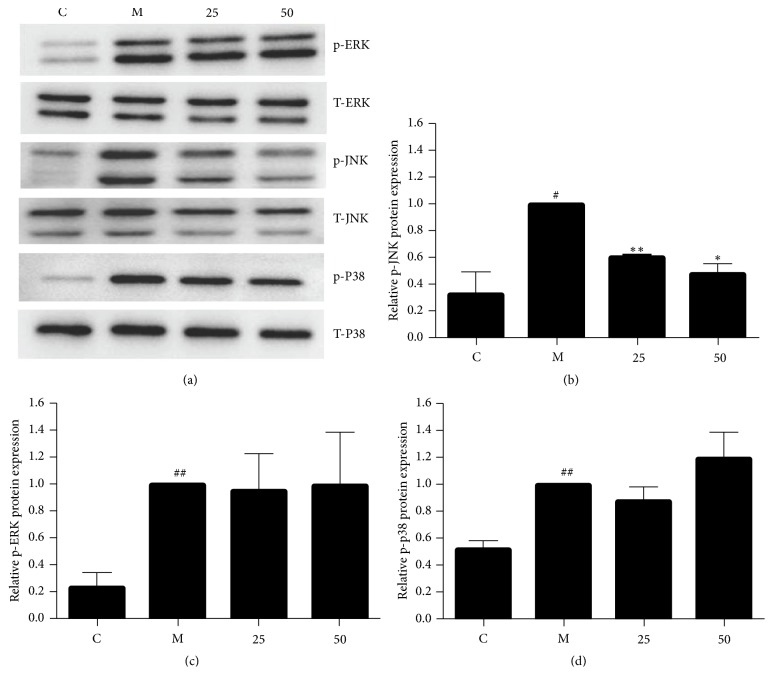
*Effect of EA11 on the MAPK signaling pathways.* (a) p-ERK, ERK, p-JNK, JNK, p-p38, and p38 protein expression was assessed by western blot following stimulation of RAW264.7 cells with M (LPS/IFN*γ* stimulation) and C (vehicle control), 25 or 50 *μ*g/ml of EA11. Band intensity of JNK (b), ERK (c), and p38 (d) was determined using an imaging densitometer band expression levels calculated relative to the intensity of total JNK/ERK/p38 protein. Values are presented as the mean ± standard deviation from three replicates. ^##^*P* < 0.01 versus C group; ^#^*P* < 0.05 versus C group; ^*∗*^*P* < 0.05 versus M group; ^*∗∗*^*P* < 0.01 versus M group.

**Figure 5:**
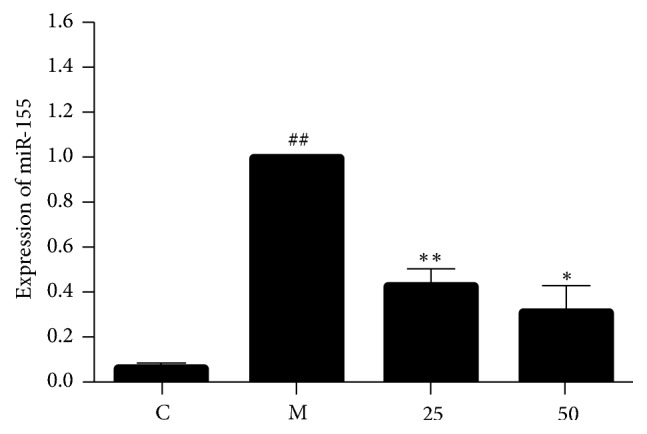
*Effect of EA11 on LPS/IFNγ-induced miR-155 levels in murine RAW264.7 cells.* RAW264.7 macrophages were incubated with pretreatment of 25 and 50 *μ*g/ml EA11 and stimulated with LPS/IFN*γ* for 24 h. Values are presented as the mean ± standard deviation from three replicates. ^##^*P* < 0.01 versus C group; ^*∗*^*P* < 0.05 versus M group; ^*∗∗*^*P* < 0.01 versus M group.

MAPK pathways are critically involved in the control of cellular responses to various cytokines and inducible synthases. To explore potential link between EA11 and MAPK signaling pathways, we determined the effect of EA11 on the phosphorylation status of ERK, JNK, and p38 in LPS/IFN*γ*-stimulated RAW264.7 cells. Western blotting showed that levels of phosphorylated ERK, p38, and JNK were all much higher in stimulated cells in comparison with unstimulated cells (Figure 4(a)). However, pretreatment of EA11 dose dependently decreased the level of phosphorylated JNK (Figure 4(b)) while displaying little effect on the phosphorylation status of p38 and ERK (Figures 4(c) and 4(d)). These results indicated that EA11 might exert its anti-inflammatory activity by the interference with JNK signaling pathway. Recent evidence has demonstrated that miR-155 is involved in inflammatory responses by serving as a master regulator to a wide range of mediators. To determine the effect of EA11 on miR-155 under the stimulation of LPS/IFN*γ* in RAW264.7 cells, we pretreated cells with EA11 prior to stimulation. qRT-PCR showed that LPS/IFN-*γ* stimulation led to a 15-fold increase in miR-155 over unstimulated cells (Figure 5). However, EA11 dramatically counteracted LPS/IFN-*γ* induced increase in miR-155 in RAW264.7 cells.

### 3.6. UPLC Analysis

**Figure 6:**
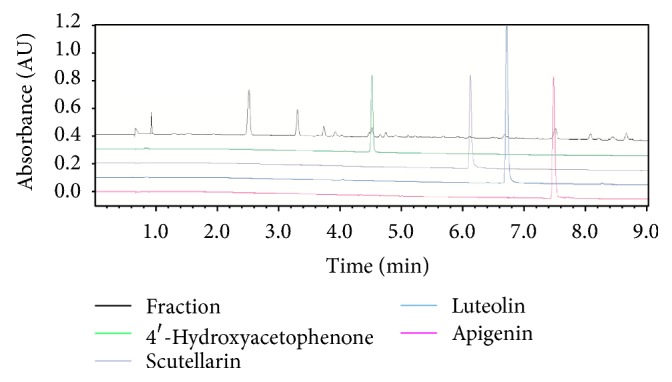
*UPLC analysis.* Four constituents: 4′-hydroxyacetophenone, scutellarin, luteolin, and apigenin.

In attempt to further understand molecular mechanism associated with EA11's anti-inflammatory activity, we characterized the constituents of EA11 UPLC analysis. Based on their UV spectra and retention time, we identified four major compounds in EA11: 4′-hydroxyacetophenone, scutellarin, luteolin, and apigenin (Figure 6), indicating that one or more of these constituents may be responsible for EA11's action.

## 4. Discussion

Herb pairs HDSB have been used in China to treat inflammation and many kinds of cancers [13, 14]. In an effort to understand the molecular mechanism associated with therapeutic functionality of HDSB, we screened fractions from HDSB for their suppressive effect on LPS/IFN*γ*-induced NO production in macrophage RAW264.7 cells. Our study showed EA11 maybe as the most potent fraction from HDSB (Table 1).

Macrophages activation is generally associated with body defense and initial immune reactions [16]. However, chronic and sustained activation of macrophage is considered to be the pathological hallmark of inflammation and has been implicated in inflammatory related diseases [17]. NO, which is produced from L-arginine by three NOS, is increased in inflammation and has proinflammatory and regulatory effects. In this study, we showed that EA11-led decrease in NO secretion was most likely mediated by reduced abundance of iNOS because EA11 greatly suppressed the expression of both iNOS mRNA and protein (Figure 2). Moreover, we also detected that EA11 also resulted in a dose-dependent reduction on IL-1*β* in LPS/IFN-*γ*-stimulated RAW 264.7 cells (Figure 3(b)), indicating that EA11 exerted its anti-inflammatory effect via different mechanisms.

Recent studies have reported that knockdown of HO-1 enhances iNOS gene expression in macrophages. Elevated level of HO-1 has also been shown to attenuate the inflammatory responses enhancing the cellular antioxidant status. The formation of bilirubin prevents the production of proinflammatory cytokine in a negative loop [18]. In this study, we showed that EA11 was able to increase the amount of HO-1 under inflammatory condition (Figure 3(a)). In addition, EA11 also upregulates the expression of PPAR-*γ*, another recognized anti-inflammatory mediator [19, 20] (Figure 3(c)). Several natural products have been demonstrated the induction of HO-1 expression in macrophages and thus eliminate the expression of proinflammatory mediators [21, 22]. EA11 has added another into this natural product list.

The MAPKs are well recognized for their role in the regulation of iNOS and IL-1*β* expression in activated macrophages [4]. ERK, JNK, and p38, three main MAPK signaling pathways, have all been shown being involved in controlling the expression of proinflammatory factors in activated macrophages. By examining the effect of EA11 on the phosphorylation status of MAPKs, we found that only the level of phosphorylated JNK was reduced effectively by EA11 (Figure 4). The specific involvement of JNK is consistent with our observation that miR-155 was downregulated by EA11 (Figure 5) because induction of miR-155 depends on NF-*κ*B and JNK signaling pathways [23, 24]. Current studies have revealed a critical role of miR-155 in inflammatory responses. We reason that EA11 at least partially acts through reducing the abundance amount of miR155 in activated macrophages.

**Figure 7:**
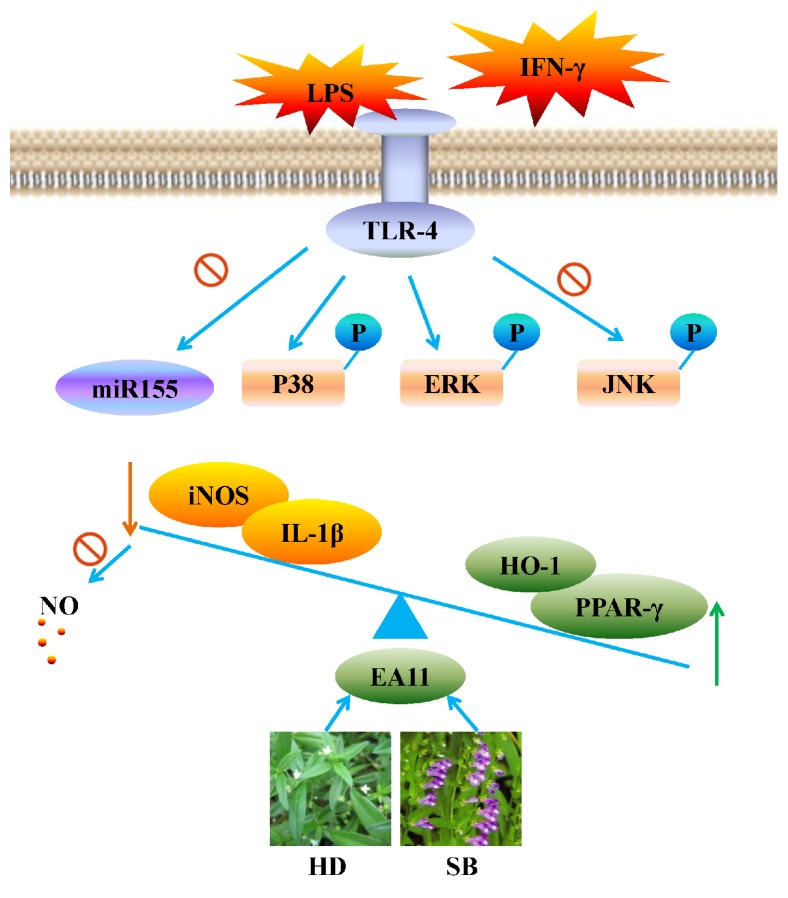
*Potential molecular mechanisms by EA11 may prevent inflammation*. EA11 may dual-regulate pro-and anti-inflammatory mediators via JNK/MAPK signal pathway and miR-155 expression to rebalance internal environment.

In the present study, we evaluated the anti-inflammatory activities of fractions from HDSB. EA11 showed the strongest activity in suppressing the nitrite content in stimulated macrophages and reduced the production of LPS/IFN-*γ* induced proinflammatory mediators such as iNOS and IL-1*β* in RAW264.7 cells. Meanwhile, EA11 increases the expression of anti-inflammatory mediators such as HO-1 and PPAR-*γ*. In the term of mechanism, the regulation of JNK/MAPK and miR-155 due to EA11 may contribute to a dual-regulation of inflammatory mediators to restore internal balance (Figure 7).

## Figures and Tables

**Table 1:** Screening extracts and fractions from HDSB on NO production (*n* = 3).

Groups	Extracts/fractions	Dose (*μ*g/ml)	Nitrite (*μ*M)	Inhibition (%)	IC_50_ (*μ*g/ml)	Yield (%)
Control	-	-	4.12 ± 0.01			
Model	-	-	53.80 ± 0.02^##^			
1 : 1 W	Water extract	10	51.79 ± 0.04^*∗*^	4.04	NC	10.45
		50	50.33 ± 0.02^*∗∗*^	6.98		
		100	50.64 ± 0.02^*∗∗*^	6.35		
		200	52.61 ± 0.03^*∗∗*^	2.39		
	Petroleum ether fraction	10	49.45 ± 0.02^*∗∗*^	8.75	52.61	0.1
		50	36.75 ± 0.03^*∗∗*^	34.31		
		100	21.14 ± 0.02^*∗∗*^	65.72		
		200	6.87 ± 0.01^*∗∗*^	94.46		
	Ethyl acetate fraction (EA11)	10	48.10 ± 0.03^*∗∗*^	11.46	27.74	0.58
		50	18.09 ± 0.02^*∗∗*^	71.87		
		100	5.98 ± 0.00^*∗∗*^	96.24		
		200	5.96 ± 0.01^*∗∗*^	96.28		
	*n*-Butanol fraction	10	48.79 ± 0.03^*∗∗*^	10.08	177	6.13
		50	40.80 ± 0.03^*∗∗*^	26.17		
		100	34.00 ± 0.02^*∗∗*^	39.86		
		200	27.63 ± 0.01^*∗∗*^	52.67		
	Water fraction	10	54.26 ± 0.02	0	685.57	30.98
		50	52.10 ± 0.04	3.43		
		100	50.45 ± 0.03^*∗∗*^	6.73		
		200	53.02 ± 0.02	1.57		
1 : 2 W	water extract	10	51.00 ± 0.03^*∗∗*^	5.63	NC	10.61
		50	46.34 ± 0.02^*∗∗*^	15		
		100	48.44 ± 0.01^*∗∗*^	10.79		
		200	45.29 ± 0.01^*∗∗*^	17.12		
	petroleum ether fraction	10	52.35 ± 0.01	2.91	128.41	0.8
		50	45.19 ± 0.06^*∗∗*^	17.33		
		100	32.75 ± 0.02^*∗∗*^	42.37		
		200	20.44 ± 0.02^*∗∗*^	67.14		
	ethyl acetate fraction	10	49.67 ± 0.04^*∗∗*^	8.3	30.2	2.11
		50	17.02 ± 0.02^*∗∗*^	74.02		
		100	6.37 ± 0.00^*∗∗*^	95.46		
		200	5.75 ± 0.00^*∗∗*^	96.71		
	n-butanol fraction	10	50.93 ± 0.02^*∗∗*^	5.77	97.09	3.81
		50	33.60 ± 0.01^*∗∗*^	40.65		
		100	28.87 ± 0.01^*∗∗*^	50.18		
		200	21.20 ± 0.01^*∗∗*^	65.61		
	water fraction	10	57.06 ± 0.02	0	456.72	58.34
		50	50.48 ± 0.00^*∗∗*^	6.67		
		100	52.44 ± 0.02	2.73		
		200	50.90 ± 0.02^*∗∗*^	5.83		
2 : 1 W	water extract	10	50.91 ± 0.03^*∗∗*^	5.82	NC	7.23
		50	49.81 ± 0.03^*∗∗*^	8.03		
		100	53.67 ± 0.02	0.25		
		200	53.00 ± 0.02	1.6		
	petroleum ether fraction	10	52.93 ± 0.03	1.75	199.67	0.03
		50	45.09 ± 0.04^*∗∗*^	17.53		
		100	40.15 ± 0.07^*∗∗*^	27.47		
		200	30.07 ± 0.05^*∗∗*^	47.76		
	ethyl acetate fraction	10	50.76 ± 0.01^*∗∗*^	6.11	44.8	1.53
		50	33.62 ± 0.01^*∗∗*^	40.62		
		100	19.55 ± 0.02^*∗∗*^	68.93		
		200	4.90 ± 0.01^*∗∗*^	98.42		
	*n*-butanol fraction	10	50.35 ± 0.02^*∗∗*^	6.93	661.8	4.62
		50	47.54 ± 0.03^*∗∗*^	12.6		
		100	40.98 ± 0.03^*∗∗*^	25.8		
		200	37.73 ± 0.02^*∗∗*^	32.34		
	water fraction	10	53.68 ± 0.04	0.24	1582.6	35.97
		50	51.54 ± 0.02^*∗*^	4.54		
		100	50.65 ± 0.03^*∗∗*^	6.33		
		200	51.38 ± 0.03^*∗*^	4.88		
	L-NIL	50 (*μ*M)	39.53 ± 0.02^*∗∗*^	35.2		

Values are presented as the mean ± standard deviation from three replicates. C, vehicle control; M, model group (LPS/IFN*γ* stimulation); EA11, ethyl acetate fraction from 1 : 1 W; ^##^*P* < 0.01 vs. C group; ^*∗*^*P* < 0.05 vs. M group; ^*∗∗*^*P* < 0.01 vs. M group.

**Table 2:** Primers used for qRT-PCR analysis (mouse).

Target gene	Primer sequence
iNOS	Forward: 5′-GGAGCGAGTTGTGGATTGTC-3′
	Reverse: 5′-GTGAGGGCTTGGCTGAGTGAG-3′
HO-1	Forward: 5′-CACAGATGGCGTCACTTCGTC-3′
	Reverse: 5′-GTGAGGACCCACTGGAGGAG-3′
IL-1*β*	Forward: 5′-GCTGTGGCAGCTACCTATGTCTTG-3′
	Reverse: 5′-AGGTCGTCATCATCCCACGAG-3′
GAPDH	Forward: 5′-AACGGATTTGGTCGTATTGGG-3′
	Reverse: 5′-CAGGGGTGCTAAGCAGTTGG-3′

## Abbreviations

EA11: Ethyl acetate extraction of* Hedyotis diffusa* Willd plus* Scutellaria barbata* D. Don
LPS: Lipopolysaccharide
IFN-*γ*: Interferon-*γ*
NO: Nitric oxide
iNOS: Inducible nitric oxide synthase
HO-1: Inducible haemoxygenase
IL-1*β*: Interleukin-1*β*
miR-155: miRNA-155
JNK: c-Jun N-terminal kinase
MAPK: Mitogen-activated protein kinase.
